# Data on serologic inflammatory biomarkers assessed using multiplex assays and host characteristics in the Multicenter AIDS Cohort Study (MACS)

**DOI:** 10.1016/j.dib.2016.08.019

**Published:** 2016-08-16

**Authors:** Heather S. McKay, Jay H. Bream, Joseph B. Margolick, Otoniel Martínez-Maza, Larry I. Magpantay, John P. Phair, Charles R. Rinaldo, Alison G. Abraham, Lisa P. Jacobson

**Affiliations:** a Department of Epidemiology, Johns Hopkins Bloomberg School of Public Health, Baltimore, MD, USA; b W. Harry Feinstone Department of Molecular Microbiology and Immunology, Johns Hopkins Bloomberg School of Public Health, Baltimore, MD, USA; c Departments of Obstetrics & Gynecology and Microbiology, Immunology & Molecular Genetics, David Geffen School of Medicine at UCLA, and Department of Epidemiology, UCLA Fielding School of Public Health, University of California, Los Angeles, CA, USA; d Northwestern University Feinberg School of Medicine, Chicago, IL, USA; e Department of Molecular Virology and Microbiology, University of Pittsburgh School of Medicine, Pittsburgh, CA, USA

## Abstract

This article contains data on the associations between fixed and modifiable host characteristics and twenty-three biomarkers of inflammation and immune activation measured longitudinally in a cohort of 250 HIV-uninfected men from the Multicenter AIDS Cohort Study (1984–2009) after adjusting for age, study site, and blood draw time of day using generalized gamma regression. This article also presents associations between each biomarker and each host characteristic in a sample restricted to 2001–2009. These data are supplemental to our original research article entitled “Host factors associated with serologic inflammatory markers assessed using multiplex assays” (McKay, S. Heather, Bream, H. Jay, Margolick, B. Joseph, Martínez-Maza, Otoniel, Phair, P. John, Rinaldo, R. Charles, Abraham, G. Alison, L.P. Jacobson, 2016) [1].

**Specifications Table**

**Table:** 

Subject area	*Epidemiology, immunology*
More specific subject area	*Cytokines, Chemokines*
Type of data	*Graphs, Heatmap*
How data was acquired	*Two electrochemiluminesence-based multiplex assay panels (Proinflammatory 9-plex and Chemokine 7-plex; Meso-Scale Diagnostics, LLC, Rockville, MD) were used to determine concentrations of IL-1β, IL-2, IL-6, IL-10, IL-12p70, GM-CSF, IFN-γ, TNF-α, CXCL8, CXCL10, CCL11, CCL2, CCL13, CCL4, and CCL17. Concentrations of sCD14, sgp130, sIL-2Rα, sTNF-R2, BAFF, sCD27, and CXCL13 were measured in a single panel (Human Biomarker Custom Premix Kit A) using the fluorescent bead-based multiplexed Luminex xMAP system at a centralized laboratory (Fluorokine*® *MAP, R&D Systems, Minneapolis, MN), and a Bio-Plex 200 Luminex instrument and Bio-Plex software (Bio-Rad, Hercules, CA). CRP was measured at Quest Diagnostics using a high-sensitivity nephelometric assay (Dade Behring, Inc., Newark, DE).*
Data format	*Analyzed*
Experimental factors	*Blood was collected at each study visit. Serum samples were processed within 6 hours of blood draw and frozen at −80* *°C. Prior to testing, a previously unthawed stock vial was thawed for each study visit, aliquoted and refrozen at −80* *°C until testing.*
Experimental features	*Specimens and data were collected from a cohort study; up to 4 visits per HIV-uninfected participant were randomly selected from individuals across the span of the study duration 1984*–*2009. All men with chronic HCV infection were included.*
Data source location	*Los Angeles, CA; Chicago, IL; Pittsburgh, PA; Washington, DC/Baltimore, MD, USA*
Data accessibility	*Data is within this article*

**Value of the data**

• These data provide previously unreported associations of a broad panel of serological inflammatory biomarker measurements with fixed and modifiable host factors in a large well-characterized cohort.
• The data provided here show the associations of host characteristics with these inflammatory biomarkers with minimal adjustment for other covariates.
• Provide estimates of association between host sociodemographics and risk behaviors with each inflammatory biomarker adjusting for co-morbidities using a dataset without missing morbidity data (restricted to participants with complete morbidity information collected 2001–2009) for comparison with the dataset that is not restricted to participants with co-morbidity information (1984–2009).
• Host factors that are associated with individual inflammatory biomarkers may be potential confounders which have implications for the design and analysis of future epidemiologic studies that seek to explore inflammatory pathways in disease pathogenesis.
• May stimulate further validation research on the relationship between these inflammatory biomarkers and host characteristics in independent populations.
• May provide insight into inflammatory disease mechanisms that can be used to identify therapeutic targets and modifiable factors, such as risk behaviors and chronic conditions.

## 1 Data

This data article is referred to in the research article entitled *Host factors associated with serologic inflammatory markers assessed using multiplex assays* [1]. We present the percent differences in distributions of biomarkers of inflammation and immune activation associated with fixed and modifiable host characteristics, minimally-adjusted for age, blood draw time of day, and study site, using serum specimens measured longitudinally (1984–2009) from a sample of HIV-uninfected men in the Multicenter Center AIDS Cohort Study (MACS). Multivariate associations between these biomarkers and sociodemographics and risk behaviors in a sample restricted to 2001–2009 and additionally adjusted for select co-morbidities were also examined.

## 2 Experimental design, materials and methods

The MACS has been previously described; briefly, it is a longstanding, prospective cohort study of men who sex with men (MSM) enrolled at four U.S. locations (Baltimore/Washington D.C., Chicago, Los Angeles, and Pittsburgh) to examine the natural and treated histories of HIV-1 infection [2], [3]. Study highlights, including data collection forms, may be found at https://statepi.jhsph.edu/macs/macs.html.

### 2.1 Biomarker data

Concentrations of 23 biomarkers of immune activation and inflammation (IL-1β, IL-2, IL-6, IL-10, IL-12p70, GM-CSF, IFN-γ, TNF-α, CXCL8, CXCL10, CCL11, CCL2, CCL13, CCL4, CCL17, sCD14, sgp130, sIL-2Rα, sTNF-R2, BAFF, and CXCL13) in archived serum specimens acquired longitudinally from 250 consenting HIV uninfected men, were obtained using two multiplex assay platforms. One additional biomarker, C-reactive protein (CRP), was measured at Quest Diagnostics (Dade Behring, Inc., Newark, DE) using a high-sensitivity immunonephelometric assay. All specimens for any given individual were tested on the same plate to minimize variability. A description of the study sample selection has been provided in our related research article [1]. Serum concentrations of IL-1β, IL-2, IL-6, IL-10, IL-12p70, IFN-ɣ, GM-CSF, TNF-α, CCL11, CXCL8, CXCL10 , CCL2, CCL13, CCL4, and CCL17 were quantitated using the Meso-Scale Discovery electrochemiluminescence-based platform (MSD; Meso-Scale Diagnostics, LLC, Rockville, MD), while concentrations of sCD14, sgp130, sIL-2Rα, sTNF-R2, BAFF, sCD27, and CXCL13 were obtained using the multiplexed Luminex xMAP system (Fluorokine^®^ MAP) using assays produced by R & D systems (Minneapolis, MN) and a Bio-Plex 200 Luminex instrument and Bio-Plex software (Bio-Rad, Hercules, CA), as described previously [1].

### 2.2 Host characteristics

The exposures of interest in the present study include sociodemographic characteristics [age at visit, race (non-black vs. black), baseline educational level (four-year college degree or higher vs. less than college degree), and body-mass index (BMI=weight (kg)/height (m)^2^; categorized as ≤24.9 (normal/underweight), 25–29.9 (overweight), and ≥30 (obese))], behavioral risk factors [smoking status (never, former, current), alcohol consumption (binge-heavy drinking [≥5 drinks/day at least once a month], moderate-heavy [3–4 drinks/day more than once a month, or ≥5 drinks/day less than once a month], low-moderate [1–2 drinks/day or 3–4 drinks/day no more than once a month], or none), and use of recreational drugs (marijuana, amyl nitrates, or any stimulant [cocaine, ecstasy, methamphetamines, or any other uppers]), hepatitis C infection (HCV) (negative, cleared [antibody+ only], or chronic [RNA positive]), presence of depressive symptoms (≥16 on the Center for Epidemiologic Studies Depression Scale (CES-D) [4]), and an expanded categorization of depressive symptoms which additionally included men who reported using medication for depression since the last visit, regardless of their CES-D score. Persistent diabetes (fasting glucose≥126 mg/dl or diabetic medication use), persistent hypertension (systolic blood pressure (SBP) ≥140 mmHg, diastolic (DBP) ≥90 mmHg, or antihypertensive medication use) were defined if present at ≥2 visits prior to blood draw. Hypercholesterolemia was present if fasting total serum cholesterol was ≥200 mg/dl. Blood draw time of day was P.M. vs. A.M.

### 2.3 Statistical analysis

Generalized gamma regression models were conducted for each biomarker to examine differences in concentrations according to categories of host characteristics described above. The generalized gamma distribution is a flexible three-parameter distribution that allows us to avoid making strong assumptions regarding the distributions of different biomarkers [5]. Biomarker concentrations were inversed so that measurements below the lower limit of detection could be handled as right-censored. In all models, only the location (β) parameter was allowed to vary by exposure category, while scale (*σ*) and shape (*λ*) were held constant. Relative percentiles (the exponential of the –β, to account for using the inverse value) in biomarker concentrations were calculated for each covariate category. Relative percentiles are presented as the percent difference in biomarker concentrations between the exposed and unexposed [(relative percentile−1) X 100]. For example, a percent difference of −50 indicates that an exposure was associated with a 50% reduction in the concentration of the biomarker compared to the unexposed. Because *σ* and *λ* were held constant, the relative percentile comparing one exposure category to another is constant across percentiles of each biomarker. Robust standard errors were adjusted for repeated measurements by using the vce(cluster) option in Stata. Age was centered at the median across all person-visits (46 years) and associations per 10-years were assessed.

**Fig. 1:**
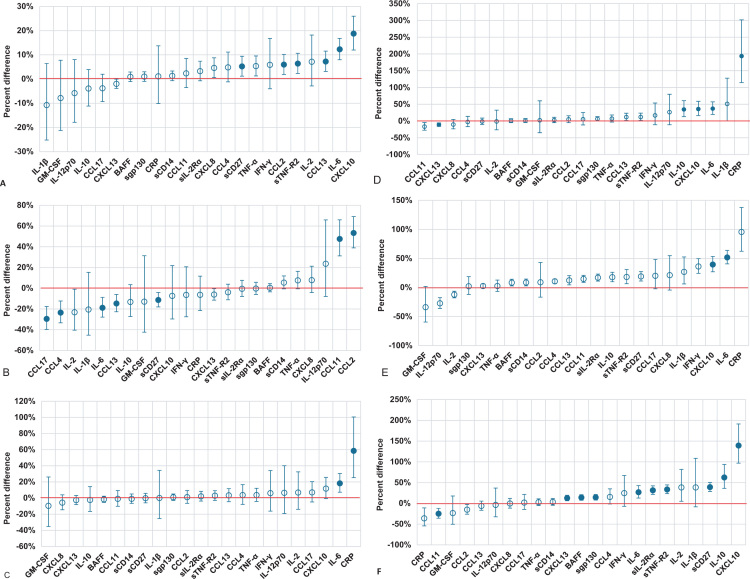

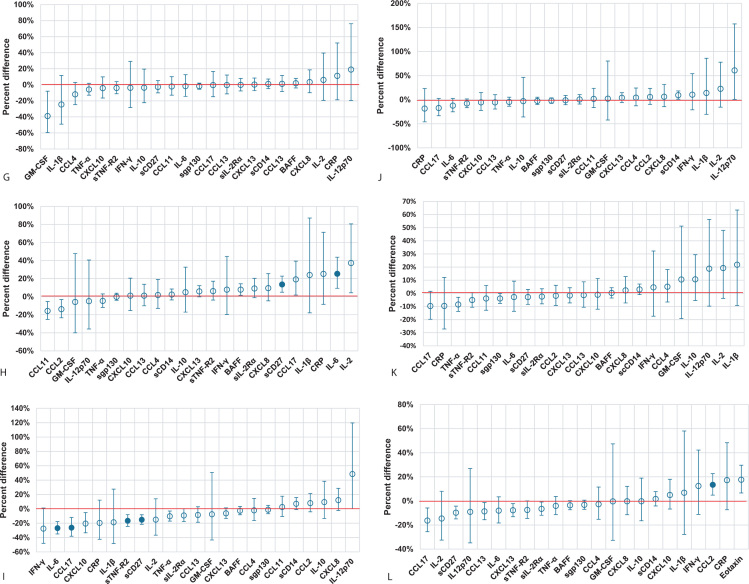

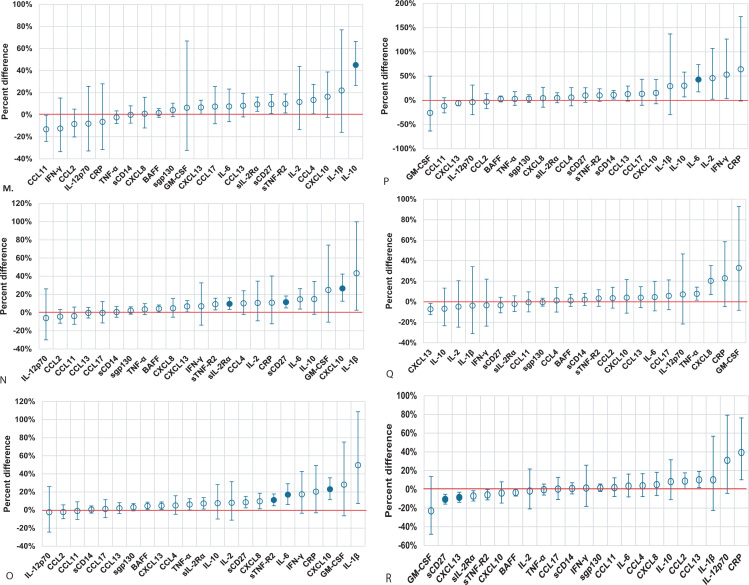
(A) Percent difference estimates for a 10-year difference in age for each biomarker, adjusted for MACS study site and time of day of blood draw. Blue circles represent the percent difference in biomarker concentrations for a 10-year difference in age. For example, a percent difference of 18 for CXCL10 indicates that a 10-year increase in age is associated with 18% higher concentrations of CXCL10. The red line indicates unity, i.e., no relative difference in biomarker concentrations. Filled markers represent statistical significance (*P*<0.002). (B) Percent difference estimates in biomarker concentrations for non-blacks vs. blacks, adjusted for age, MACS study site, and time of day of blood draw. Blue circles represent the percent difference in biomarker concentrations for non-blacks relative to blacks. The red line indicates unity, i.e., no relative difference in biomarker concentrations. Filled markers represent statistical significance (*P*<0.002). (C) Percent difference estimates in biomarker concentrations for overweight BMI vs. normal BMI, adjusted for age, MACS study site, and time of day of blood draw. Blue circles represent the percent difference in each biomarker concentration between men with overweight BMI relative to men with normal BMI. The red line indicates unity, i.e., no relative difference in biomarker concentrations. Filled markers represent statistical significance (*P*<0.002). (D) Percent difference estimates in biomarker concentrations for obese BMI vs. normal BMI, adjusted for age, MACS study site, and time of day of blood draw. Blue circles represent the percent difference in biomarker concentrations for men with obese BMI relative to men with normal BMI. The red line indicates unity, i.e., no relative difference in biomarker concentrations. Filled markers represent statistical significance (*P*<0.002). (E) Percent difference estimates in biomarker concentrations for those with cleared hepatitis C infection (antibody+ only) vs. those who are not infected with hepatitis C, adjusted for age, MACS study site, and time of day of blood draw. Blue circles represent the percent difference in biomarker concentrations for men with cleared hepatitis C infection (antibody + only) relative to those without hepatitis C infection. The red line indicates unity, i.e., no relative difference in biomarker concentrations. Filled markers represent statistical significance (*P*<0.002). (F) Percent difference estimates in biomarker concentrations for those with chronic hepatitis C infection vs. those who are not infected with hepatitis C, adjusted for age, MACS study site, and time of day of blood draw. Blue circles represent the percent difference in biomarker concentrations for men with chronic HCV relative to those without HCV. The red line indicates unity, i.e., no relative difference in biomarker concentrations. Filled markers represent statistical significance (*P*<0.002). (G) Percent difference estimates in biomarker concentrations for former smokers vs. never smokers, adjusted for age, MACS study site, and time of day of blood draw. Blue circles represent the percent difference in biomarker concentrations for former smokers relative to never smokers. The red line indicates unity, i.e., no relative difference in biomarker concentrations. Filled markers represent statistical significance (*P*<0.002). (H) Percent difference estimates in biomarker concentrations for current smokers vs. never smokers, adjusted for age, MACS study site, and time of day of blood draw. Blue circles represent the percent difference in biomarker concentrations for current smokers relative to never smokers. The red line indicates unity, i.e., no relative difference in biomarker concentrations. Filled markers represent statistical significance (*P*<0.002). (I) Percent difference estimates in biomarker concentrations for moderate-heavy alcohol consumption vs. no alcohol consumption since last visit, adjusted for age, MACS study site, and time of day of blood draw. Blue circles represent the percent difference in biomarker concentration for moderate-heavy alcohol consumption relative to no alcohol consumption. The red line indicates unity, i.e., no relative difference in biomarker concentrations. Filled markers represent statistical significance (*P*<0.002). (J) Percent difference estimates in biomarker concentrations for binge alcohol consumption vs. no alcohol consumption since last visit, adjusted for age, MACS study site, and time of day of blood draw. Blue circles represent the percent difference in biomarker concentration for binge alcohol consumption relative to no alcohol consumption. The red line indicates unity, i.e., no relative difference in biomarker concentrations. Filled markers represent statistical significance (*P*<0.002). (K) Percent difference estimates in biomarker concentrations for marijuana use vs. no marijuana use since last visit, adjusted for age, MACS study site, and time of day of blood draw. Blue circles represent the percent difference in biomarker concentration for use of marijuana vs. no use of marijuana. The red line indicates unity, i.e., no relative difference in biomarker concentrations. Filled markers represent statistical significance (*P*<0.002). (L) Percent difference estimates in biomarker concentrations for use of amyl nitrates vs. no use of amyl nitrates since last visit, adjusted for age, MACS study site, and time of day of blood draw. Blue circles represent the percent difference in biomarker concentrations for use of amyl nitrates relative to no use of amyl nitrates. The red line indicates unity, i.e., no relative difference in biomarker concentrations. Filled markers represent statistical significance (*P*<0.002). (M) Percent difference estimates in biomarker concentrations for use of stimulants vs. no use of stimulants since last visit, adjusted for age, MACS study site, and time of day of blood draw. Blue circles represent the percent difference in biomarker concentration for use of stimulants relative to no use of stimulants. The red line indicates unity, i.e., no relative difference in biomarker concentrations. Filled markers represent statistical significance (*P*<0.002). (N) Percent difference estimates in biomarker concentrations for depressive symptoms, adjusted for age, MACS study site, and time of day of blood draw. Blue circles represent the percent difference in biomarker concentrations for presence of depressive symptoms (CES-D ≥ 16 only) relative to no presence of depressive symptoms. The red line indicates unity, i.e., no relative difference in biomarker concentrations. Filled markers represent statistical significance (*P*<0.002). (O) Percent difference estimates in biomarker concentrations for depression, adjusted for age, MACS study site, and time of day of blood draw. Blue circles represent the percent difference in biomarker concentrations for depression (CES-D≥16 or self-reported use of depression medication) relative to no depression. The red line indicates unity, i.e., no relative difference in biomarker concentrations. Filled markers represent statistical significance (*P*<0.002). (P) Percent difference estimates in biomarker concentrations for persistent diabetes, adjusted for age, MACS study site, and time of day of blood draw. Blue circles represent the percent difference in biomarker concentrations for men with persistent diabetes relative to those without persistent diabetes. The red line indicates unity, i.e., no relative difference in biomarker concentrations. Filled markers represent statistical significance (*P*<0.002). (Q) Percent difference estimates in biomarker concentrations for persistent hypertension, adjusted for age, MACS study site, and time of day of blood draw. Blue circles represent the percent difference in biomarker concentrations for men with persistent hypertension relative to those without persistent hypertension. The red line indicates unity, i.e., no relative difference in biomarker concentrations. Filled markers represent statistical significance (*P*<0.002). (R) Percent difference estimates in biomarker concentrations for hypercholesterolemia, adjusted for age, MACS study site, and time of day of blood draw. Blue circles represent the percent difference in biomarker concentration for men with hypercholesterolemia relative to men without hypercholesterolemia. The red line indicates unity, i.e., no relative difference in biomarker concentrations. Filled markers represent statistical significance (*P*<0.002) (For interpretation of the references to color in this figure legend, the reader is referred to the web version of this article).

Fig. 1 shows the estimated percent differences in each biomarker by host characteristic exposure status, minimally-adjusted for age, study site, and blood draw time of day and the full sample of person-visits (1984–2009).

**Fig. 2:**
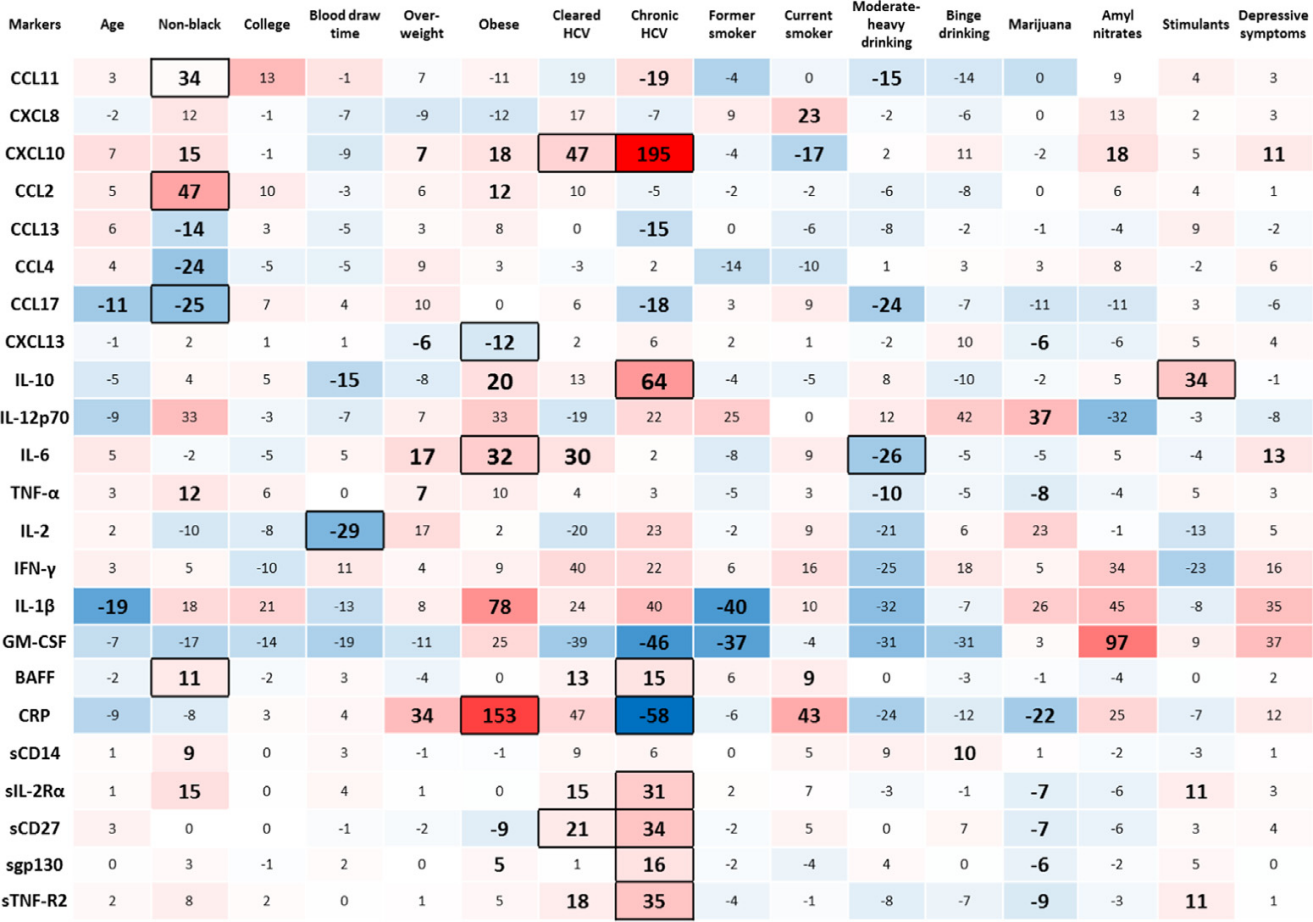
Percent differences from multivariate generalized gamma regression models examining the associations of age (10-year), non-black race, college education at baseline, blood draw time of day (P.M. versus A.M.), being overweight, obesity, cleared (antibody + only) hepatitis C infection (HCV), chronic hepatitis C infection, former smoking, current smoking, moderate-heavy alcohol consumption, binge alcohol consumption, use of marijuana, use of amyl nitrates, use of stimulants, the presence of depressive symptoms, and depression including those taking depression medication (*column headings*) with individual biomarkers (*row headings),* adjusting for persistent diabetes, persistent hypertension, and hypercholesterolemia, in a sample restricted to 2001–2009. Large **bold** text **with** black-bordered cells indicates significance at the *P<*0.002 level; large **bold** text **without** borders indicates marginal significance (0.002<*P*≤0.05). The color gradient of each cell illustrates the magnitude of the estimate (darker red indicating stronger positive percent difference and darker blue indicating stronger negative percent difference). For example, chronic HCV is associated with 64% higher concentrations of IL-10 (*P*<0.002) and 19% lower concentrations of CCL11 (0.002<*P*≤0.05).

Fig. 2 shows the estimated associations between host characteristics and each biomarker from models fully-adjusted for all sociodemographics and risk behaviors, and the select morbidities (persistent diabetes, persistent hypertension, and hypercholesterolemia) in a sample restricted to 2001–2009, when consistent ascertainment of data on these morbidities began in the MACS. The estimated percent differences in Fig. 2 are presented in the form of a heatmap, which allows examination of the associations between individual characteristics with each biomarker as well as examining the biomarkers that are associated with each exposure. Color densities for the heatmap are standardized using the sample standard deviation of each exposure.

All statistical tests were 2-sided. To account for multiple testing, a Bonferroni adjustment at an α-level of 0.05 [(0.05/23)=0.002] for statistical significance was employed. Estimates with 0.002<*P*≤0.05 were considered marginally significant.

Data were analyzed using SAS, version 9.3 (SAS Institute, Inc., Cary, North Carolina) and Stata, version 13 (College Station, Texas).
